# Supplementary material for: Monkeypox Vaccine Acceptance among Ghanaians: A Call for Action
Source: Vaccines (Basel). 2023 Jan 21;11(2):240. doi: 10.3390/vaccines11020240 (PMC9959510; doi:10.3390/vaccines11020240)
Supplement: Supplementary file 1 [file vaccines-11-00240-s001.zip › vaccines-2125208-supplementary.pdf]

Monkeypox vaccine acceptance among Ghanaians: a call for action

Questionnaire

You are invited to participate in a paper entitled "Evaluation of Psychological Drivers of Monkey Pox Virus Vaccines in Ghana". This research aims to assess the public's beliefs regarding the monkeypox vaccine. This research is aimed at the general public and healthcare workers over the age of 18 years. The results of the current research may provide an opportunity to develop an awareness plan about the monkeypox vaccine in order to enhance knowledge and dispel myths about it. Your participation in this survey is voluntary and you are free to withdraw from this study at any time you wish. This study has been approved by the College of Medicine Ethics Committee No. 00018699 and all data will be anonymized without any indication of your personal identity.

Monkeypox, a disease that is spreading in the world We, as doctors and researchers from several universities, hospitals and medical centers, invite you to participate in this study, which aims to assess the psychological motives for vaccinations against monkeypox.

Consent question:

- **Participate in the survey**
  - Agree
  - Disagree

Section 1: Socio-demographics questions:

- **Gender**
  - Male
  - Female
- **Age in years …….**
- **Nationality …….**
- **Country where you are living now: …….**
- **Marital status:**
  - Single
  - Married
  - Having partner
  - Widow
- **Living area:**
  - Urban
  - Rural
  - Other: ……
- **Region:**
  - Ahafo

- Ashanti
- Bono east
- Brong Ahafo
- Central
- Eastern
- Greater Accra
- North east
- Northern
- Oti
- Savannah
- Upper east
- Upper west
- Western
- Western north
- Volta

● **Financial status:**
- Low income
- Middle income
- Upper income

● **What is the highest level of education/degree you have completed?**
- Pre-college/High school
- Professional/technical
- Bachelor
- Diploma
- Postgraduate (Master)
- Postgraduate (PhD)

● **Do you suffer from any chronic diseases?**
- Yes
- No

● **Occupation**
- Physician /Physician Assistant
- Nurse/midwife
- Pharmacist/ pharmacy technician
- Laboratory technician
- Public health personnel
- Other (not in the healthcare fi eld)
- Student

Section II

• **Did you receive Covid-19 vaccine**
- Fully vaccinated
- took first dose, going to take the second
- took first dose, will not take the second
- not going to take the vaccine

● **Have you had Monkeypox?**
- Yes
- No

- I don't know

- **Do you know anyone died due to Monkeypox, that you know?**
  - Yes
  - No
  - I don't know
- **Do you intend to get Monkeypox vaccination?**
  - Yes
  - No

<u>Section III: Please answer these questions about Monkeypox vaccinations:</u>

- **I am completely confident that vaccines are safe**
  - strongly agree
  - agree
  - neutral
  - disagree
  - strongly disagree
- **Vaccinations are effective**
  - strongly agree
  - agree
  - neutral
  - disagree
  - strongly disagree
- **Regarding vaccines, I am confident that public authorities decide in the best interest of the community**
  - strongly agree
  - agree
  - neutral
  - disagree
  - strongly disagree
- **Vaccination is unnecessary because vaccine preventable diseases are not common any more**
  - strongly agree
  - agree
  - neutral
  - disagree
  - strongly disagree
- **My immune system is so strong, it also protects me against diseases**
  - strongly agree
  - agree
  - neutral
  - disagree
  - strongly disagree
- **Vaccine-preventable diseases are not so severe that I should get vaccinated**
  - strongly agree
  - agree
  - neutral
  - disagree

- strongly disagree

- **Everyday stress prevents me from getting vaccinated**
  - strongly agree
  - agree
  - neutral
  - disagree
  - strongly disagree
- **For me, it is inconvenient to receive vaccinations**
  - strongly agree
  - agree
  - neutral
  - disagree
  - strongly disagree
- **Visiting the doctors' makes me feel uncomfortable; this keeps me from getting vaccinated**
  - strongly agree
  - agree
  - neutral
  - disagree
  - strongly disagree
- **When I think about getting vaccinated, I weigh benefits and risks to make the best decision possible**
  - strongly agree
  - agree
  - neutral
  - disagree
  - strongly disagree
- **For each and every vaccination, I closely consider whether it is useful for me**
  - strongly agree
  - agree
  - neutral
  - disagree
  - strongly disagree
- **It is important for me to fully understand the topic of vaccination, before I get vaccinated**
  - strongly agree
  - agree
  - neutral
  - disagree
  - strongly disagree
- **When everyone is vaccinated, I don't have to get vaccinated too**
  - strongly agree
  - agree
  - neutral
  - disagree
  - strongly disagree
- **I get vaccinated because I can also protect people with a weaker immune system**

- strongly agree
- agree
- neutral
- disagree
- strongly disagree

- **Vaccination is a collective action to prevent the spread of disease**
    - strongly agree
    - agree
    - neutral
    - disagree
    - strongly disagree

THANK YOU FOR PARTICIPATION
